# Supplementary material for: Human stem cell models for group 3 medulloblastoma uncover JARID1B as a regulator of the chromatin landscape
Source: bioRxiv. 2025 Dec 8:2025.12.06.689939. Preprint. [Version 1] doi: 10.64898/2025.12.06.689939 (PMC12709477; doi:10.64898/2025.12.06.689939)
Supplement: Supplement 3 [file media-3.pdf]

Qadeer et al., Figure S3

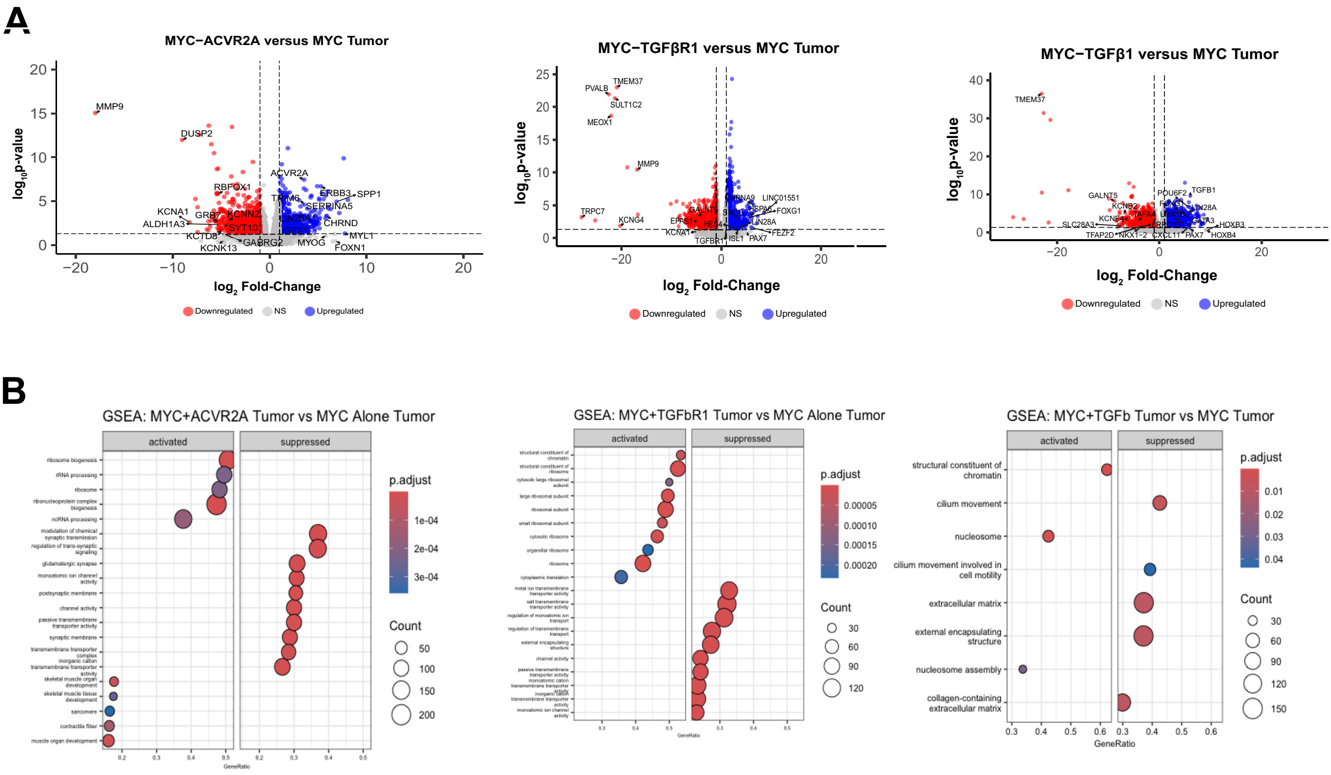

Figure S3. RNA-seq analysis of MYC-TGFβ driven NESC-derived tumors. A, Volcano plots comparing differentially expressed genes (DEG) between NESC-derived tumors driven by MYC-TGFβ versus MYC alone. Significant DEGs were called with log2FC ≥1 for Upregulated and log2FC ≤1 and Downregulated, p<0.05. **B**, Gene set enrichment analysis (GSEA) of significant DEGs for each genotype using clusterProfiler.
