# Supplementary material for: Human stem cell models for group 3 medulloblastoma uncover JARID1B as a regulator of the chromatin landscape
Source: bioRxiv. 2025 Dec 8:2025.12.06.689939. Preprint. [Version 1] doi: 10.64898/2025.12.06.689939 (PMC12709477; doi:10.64898/2025.12.06.689939)
Supplement: Supplement 5 [file media-5.pdf]

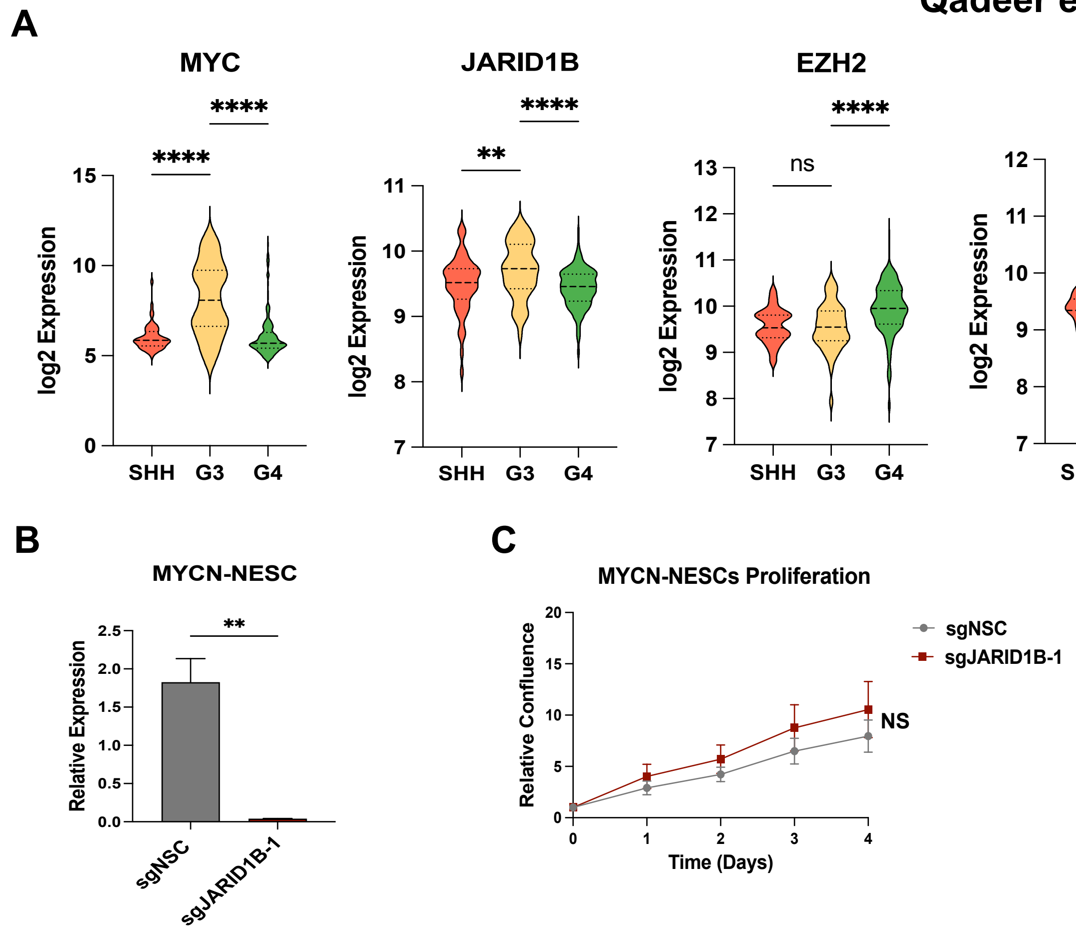

Figure S5. JARID1B analysis in human MB and NESC cell lines. A, Boxplot of expression of PRC-interacting genes in MB subgroups in the MAGIC dataset. **B**, qRT-PCR analysis for JARID1B in MYCN-expressing NESCs. Relative expression normalized to GAPDH, mean± SEM (n=3) **C**, Cell proliferation of MYCN-NESC control and JARID1B knockdown cells over 5 days. Percent confluence normalized to D0 for each line, average mean± SEM (n=3). Statistical signifance calculated using unpaired student's t-test or one-way ANOVA with Tukey's multiple comparision test. (*, p<0.05, **, p<0.01; ***, p<0.001, ****, p<0.0001)
